# 

**Published:** 1858-12

**Authors:** 


### RECEIPTS FOR JOURNAL UP TO JANUARY 1st, 1859.

### Illinois.

| | | |
|---|---|---|
| Dr. T. B. Dever, | vol. 1. 1858. | $2 |
| " John Allen, | " " | 2 |
| " J. Welsh, | " " | 2 |
| " C. N. Irwin, | " " | 2 |
| " M. Wiley, | " " | 2 |
| " Askew & Mitchell, | " " | 2 |
| " J. S. Dewey, | half of vol. 3 & 4, 1854-5, | 3 |
| " R. F. Carr, | vols. 5, 6 & 1, 1856-7-8, | 5 |
| " C. W. Knott, | " " " | 6 |
| " Alex. Hull, | " 6, 1 & 2, 1857-8-9, | 6 |
| " Geo. V. Ewing, | vol. 2, 1859, | 2 |
| " C. Goodbrake, | " " | 2 |

### Indiana.

| | | |
|---|---|---|
| Dr. J. J. Thompson, | vol. 1, 1858, | $2 |
| " Geo. W. Slack, | vols. 4, 5, 6, 1, 1855-6-7-8, | 8 |
| " N. H. Canaday, | vol. 2, 1859, | 1 |

### Iowa.

| | | |
|---|---|---|
| Dr. W. A. Morse, | vol. 1, 1858, | $2 |
| " C. W. Davis, | " " | 2 |
| " J D. Blanchard, | half of vol. 5 & 6, 1856-7, | 3 |

### Wisconsin.

| | | |
|---|---|---|
| Dr. O. D. Coleman, | vol. 1, 1858, | $2 |

